# Enhancement of the Quality of the Shell-Core Bond Interface in Duplex Work Rolls Manufactured by Centrifugal Casting Used in Hot Strip Mills

**DOI:** 10.3390/ma12081304

**Published:** 2019-04-20

**Authors:** Alberto Cofiño-Villar, Florentino Alvarez-Antolin, Juan Asensio-Lozano

**Affiliations:** Materials Pro Group, Departamento de Ciencia de los Materiales e IngenieríaMetalúrgica, Universidad de Oviedo; Independencia 13, 33004 Oviedo, Spain; UO229780@uniovi.es (A.C.-V.); jasensio@uniovi.es (J.A.-L.)

**Keywords:** shell-core bond, work rolls, centrifugal casting, hot strip mill, Cr_3_C, NbC

## Abstract

To ensure the formation of a sound shell-core bond interface free of defects between the shell and the core in work rolls used in the finishing stands of hot strip mills, a complete fusion of this interface must be achieved, avoiding excessive mixing of the two components and the formation of hard, fragile microstructures. The shell is made of white cast iron, alloyed with Ni and Cr, and the core is manufactured of grey cast iron spheroidal graphite in a pearlitic matrix. It is thus advisable to inoculate the shell with 0.6 kg/T SiCaMn, as this promotes discontinuity in the carbide network and leads to an increase in the impact toughness of the bond interface. Furthermore, inoculation of the shell with FeSi-La should be avoided, as this inoculant leads to an increase in graphite counts, promoting it with a lamellar morphology at the edge of the bond and hence reducing the impact toughness in this interface. Addition of Mg to the shell has been found to produce an increase in hardness in the regions adjacent to the bond interface.

## 1. Introduction

Indefinite chill double-poured (ICDP) work rolls are used in the finishing stands of hot strip mills (HSMs). These rolls usually comprise an outer shell made of cast iron, alloyed with Ni and Cr, and a grey cast iron core containing spheroidal graphite in a mostly pearlitic matrix. The term “indefinite” refers to the fact that part of the carbon in the shell appears in the form of carbides, while another, small part appears in the form of graphite [1]. The graphite performs dry lubrication functions [2] and reduces the coefficient of friction during the rolling process [3]. In the early phases of the hot strip mill, during the rolling passes, the shell or working layer is subjected to severe thermal fatigue cycles, reaching over 500 °C when it comes into contact with the steel strip and cooling down to 50 °C via the application of jets of water at the beginning and end of the rolling pass [4,5]. The presence of graphite facilitates the evacuation of heat due to its high thermal conductivity [6,7,8], flake graphite being more favorable for this purpose than graphite with a spheroidal morphology [9,10], although the latter improves mechanical strength [11]. During each rolling pass, the roll is thus subjected to compression forces that oppose thermal expansion. The majority of carbides will be formed with Cr with a stoichiometry of M_3_C, whose hardness can reach 1200 HV [12]. The presence of mixed carbides in a martensitic matrix ensures high wear resistance [13].To enhance wear resistance, Nb and Mo can be added, which form carbides with a stoichiometry of MC, obtaining hardness values that can reach 2400 HV [14].The Ni and almost all the Mo will be in solid solution to increase the hardenability of the austenite [15], which is mostly transformed into martensite after a heat treatment at 1000 °C and air cooling. These rolls are manufactured by centrifugal casting [16,17,18,19,20,21]. First, the shell of the roll (outer working layer) is cast, and once this outer shell has solidified, the core is cast in two stages. In the first stage, the part of the core intended to achieve an optimal bond with the shell is cast. This intermediate layer is necessary to create a good bond between the working layer and the core. Subsequently, the remainder of the core and roll necks are casted by gravity and solidify statically. The bonding between the working layer and the core is important since poor bonding can cause bond-related spalls [22]. Demolding takes place 4 or 5 days after casting. After heat treatment at 1000 °C with air cooling, the roll is subjected to tempering at 400 °C. To ensure the formation of a sound bond interface free of defects, complete bonding of the shell-core interface must be achieved, avoiding excessive mixing of the two components [23]. This bonding region will inevitably be contaminated with alloy elements, such as Cr, resulting in a microstructure of grey cast iron that combines spheroidal graphite and ledeburite [24]. During the casting process and the slow cooling during solidification until demolding, diffusive phenomena take place between the shell and the core, especially among the alloy elements of the shell towards the core. Hard, fragile microstructures may be formed in the shell-core bonding region that might weaken this bond when subjected to the pressures exerted during the rolling pass. The presence of non-metallic inclusions or microcavities can also adversely affect this bond. All this can lead to fracture and spalling between the shell and the core [25]. Employing a design of experiments (DOE), the aim of this paper is to identify those manufacturing factors involved in the manufacture of the shell that might have a significant effect on the quality of the shell-core interface. The impact toughness test is a reliable test method for assessing the quality of this bi-metal bond [26,27,28]. The analyzed factors were: The use of FeSi alloy inoculants with traces of lanthanum; inoculation with different percentages of SiCaMn, FeB, and Mg; the percentage of Si; and the liquidus temperature. Previous studies by the authors have analyzed the influence of these factors on the mechanical and microstructural properties of the working layers [29,30]. The present paper analyzes their effect on the shell-core bond interface in work rolls.

## 2. Materials and Methods 

Table 1 shows the most usual chemical composition range for both the shell and the core of the work roll.

The experimental procedure employed is based on the design of experiments (DOE) statistical technique [31]. The purpose of applying this technique is to modify certain normal working conditions deliberately so as to produce changes in some of the responses under study. In this case, deliberate changes in six industrial manufacturing factors were analyzed, carrying out eight experiments in all, at an industrial scale. Table 2 shows the analyzed factors and levels, while Table 3 displays the resulting array of experiments. The set of generators associated with this array of experiments is D = AB, E = AC and F = BC. This means that the interactions ABD, ACE, and BCF are confounded with the mean.

The resolution of this design of experiments is III, which means that the main effects are confounded with the interactions of two factors [31]. The effect of a factor is the variation in the response function as a consequence of the variation of said factor. These effects are defined as the main effects. The effect of one factor may often depend on the value that another takes. When this occurs, these factors are said to interact. The importance of the main effects tends to be greater than that of the interactions of two factors, while the importance of the latter is in turn greater than that of the interactions of three factors, and so on. The “Confounding Pattern” column in Table 3 indicates those second-order interactions whose effects are confounded with the main effects. For example, the effect of the second-order interactions BD and CE will be confounded with the effect of factor A [31]. In this design of experiments (DOE), we analyze six factors, with two levels for each factor. Thus, if the DOE were not fractional, it would be necessary to estimate 26 effects (64 effects) in all. However, as the DOE is fractional, we only estimate eight effects (2^6−3^): Hence, seven other effects are confounded in each effect (64/8 = 8). The confounding pattern should include all the effects confounded with one another. However, given that the number of confounding patterns is very high (8), Table 3 shows a restricted confounding pattern in which only the main effects and the two-factor interactions are represented.

The experimental response is subject to random variation. This variation will follow a normal law, where its standard deviation reflects experimental error. The effects are linear combinations of the responses. Hence, applying the central limit theorem (CLT), they follow a normal law. Each main effect may be considered a random variable where the obtained value is an estimate of its mean. If all the effects were non-significant, they would follow an N(0,σ) law and would thus appear aligned in a representation of the effects on a normal probability plot. If any effect is significant, it will follow an N(μ,σ) law, not appearing aligned with the non-significant effects [31]. The standardized effect is the ratio between the difference in the value of the response of each experiment and the mean value of all the experiments and its standard deviation. This represents not only whether the value of the variable is above or below the mean, but also how far it deviates from it. Those standardized effects that deviate from the straight line towards the ends on the normal probability plot are significant. Those that deviate to the left indicate that the value of the response increases at their −1 level, while, analogously, those that deviate to the right indicate that the value of the response increases at their +1 level.

Table 4 shows the chemical composition of the inoculants used to manufacture the shell. The spheronization treatment was carried out in a covered ladle. The procedure consisted of introducing a 13 mm wire into the liquid alloy through an opening in the cover. The wire alloy was FeSiMg.

The research was conducted at an industrial scale, for which purpose eight rolls with a diameter between 680 and 700 mm, a length between 1800 and 2000 mm, and a thickness of the working layer between 50 and 55 mm were cast. Table 5 provides the casting parameters of each layer in the eight experiments.

Three unnotched Charpy specimens measuring 10 mm × 10 mm × 50 mm were extracted from the bonding region between the shell and the core of the eight work rolls, the shell-core interface being parallel to the longest dimension. Accordingly, the direction of impact with the Charpy pendulum (HOYTOM, Leioa, Spain) was perpendicular to this bond interface. Three Charpy tests were performed for each experiment. Furthermore, a Vickers hardness profile was determined in each of the Charpy specimens, thereby enabling the transition between the shell and the core to be described. A load of 0.5 kg was applied for this purpose. In all, 23 Vickers indentations with a length of 8.8 mm were made in each Charpy specimen, with a separation of 400 μm between each indentation. The hardness profile of each experiment corresponds to the mean values of three measurements.

The factors with significant influence on the following responses were determined by means of the DOE:

1. Impact toughness using a Charpy pendulum.
2. Hardness of the part of the shell adjacent to the bond edge. To obtain this value, the hardness data obtained at a thickness of 2 mm from this edge were considered.
3. Hardness of the part of the core adjacent to the bond edge. Likewise, the hardness data obtained at a thickness of 2 mm from this edge were considered to obtain this value.

The optical microscope employed was a NIKON Epiphot 200 (Nikon, Tokyo, Japan), and the different types of precipitated carbides were identified under a JEOL JSM-5600 (JEOL, Nieuw-Vennep, The Netherlands) scanning electron microscope (SEM), equipped with the characteristic X-ray scattering microanalysis system (SEM-EDX).

## 3. Results

**Figure 1:**
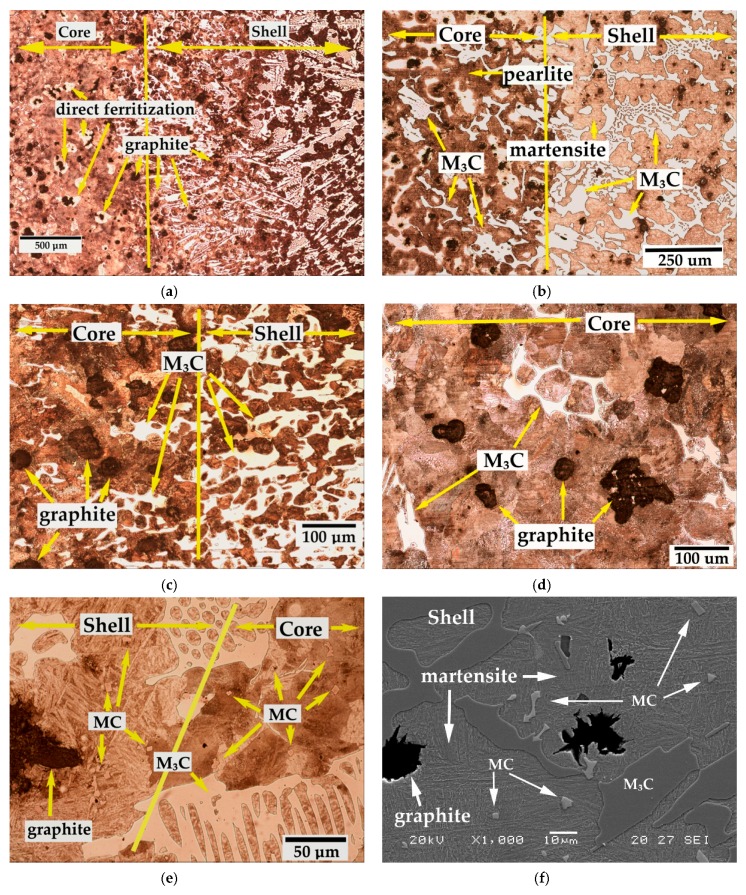
Microstructure of the shell-core bonding region. (**a**) Bonding region corresponding to experiment 2. Ferrite regions can be observed around the spheroidal graphite. Micrograph obtained by optical microscopy. ×50 magnifications, (**b**) Bonding region corresponding to experiment 3. Martensite is the matrix phase in the part of the bond corresponding to the shell and pearlite is the constituent matrix of the bond in the part of the core. Micrograph obtained by optical microscopy. ×100 magnifications, (**c**) Bonding region corresponding to experiment 2. The penetration of M_3_C carbides towards the core is observed. Micrograph obtained by optical microscopy. ×200 magnifications, (**d**) Bonding region corresponding to experiment 8. M_3_C carbides are observed in the part of the core adjacent to the bond. Micrograph obtained by optical microscopy. ×200 magnifications, (**e**) Bonding region corresponding to experiment 1. MC (NbC) carbides are observed in the core. Micrograph obtained by optical microscopy. ×500 magnifications, (**f**) Bonding region corresponding to experiment 7. Presence of mixed carbides in a martensitic matrix. Micrograph obtained by scanning electron microscopy. ×1000 magnifications.

Figure 1 shows representative images of the microstructure in the shell-core bonding region. The appreciable penetration of M_3_C carbides towards the core is worth noting. The matrix phase in the part of the bond corresponding to the shell is martensite, while the constituent matrix of the bond in the part of the core is pearlite. In this latter part, ferrite regions (direct ferritization) can be observed around the spheroidal graphite. Figure 1e,f shows the presence of MC (NbC) carbides at the edge of the bond, even appearing in the core, in the region adjacent to the bond.

Table 6 shows the mean values obtained for the studied responses and the standardized effects corresponding to the factors and interactions indicated in the column denominated “Confounding Pattern”. The row corresponding to the mean shows the average value obtained for each of the responses in the eight experiments.

**Figure 2:**
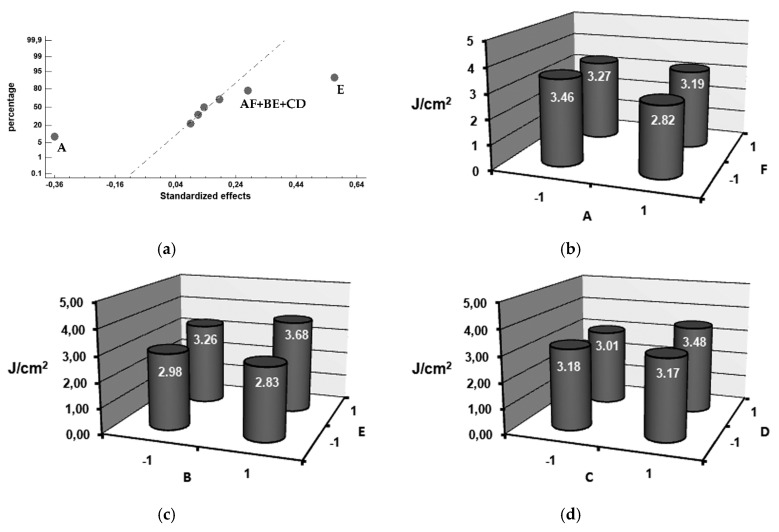
Factors that have a significant effect on impact toughness in the bond interface. (**a**) Standardized effects on a normal probability plot, (**b**) Analysis of interaction AF, (**c**) Analysis of interaction BE, (**d**) Analysis of interaction CD.

**Figure 3:**
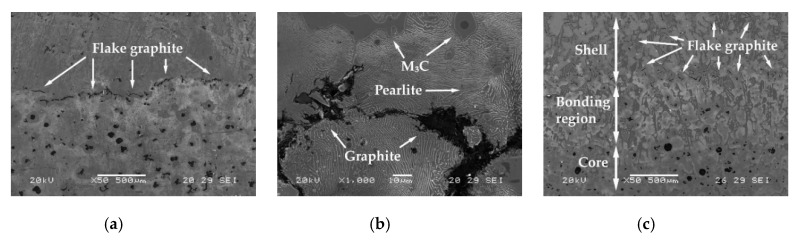
Flake graphite located in the region of the shell adjacent to the bond interface. This mainly appears in those experiments in which the shell was inoculated with FeSi-La, (**a**) Bonding region corresponding to experiment 2. Micrograph obtained by scanning electron microscopy. ×50 magnifications, (**b**) Bonding region corresponding to experiment 2. In addition to the presence of flake graphite, the presence of eutectic M_3_C carbides and austenite fully transformed into pearlite can also be observed. Micrograph obtained by scanning electron microscopy. ×1000 magnifications, (**c**) Bonding region corresponding to experiment 4. Micrograph obtained by scanning electron microscopy. ×500 magnifications.

**Figure 4:**
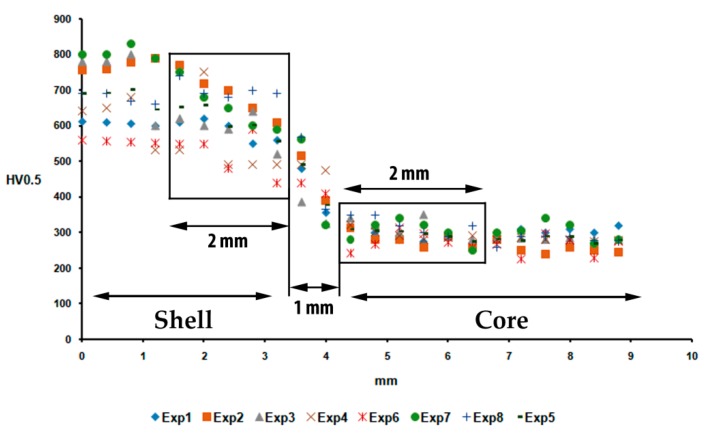
Hardness profiles in the shell-core bonding region. The statistically analyzed data were collected from a width of 2 mm in the regions of the shell and the core adjacent to the bond interface between both. Exp (Experiment).

**Figure 5:**
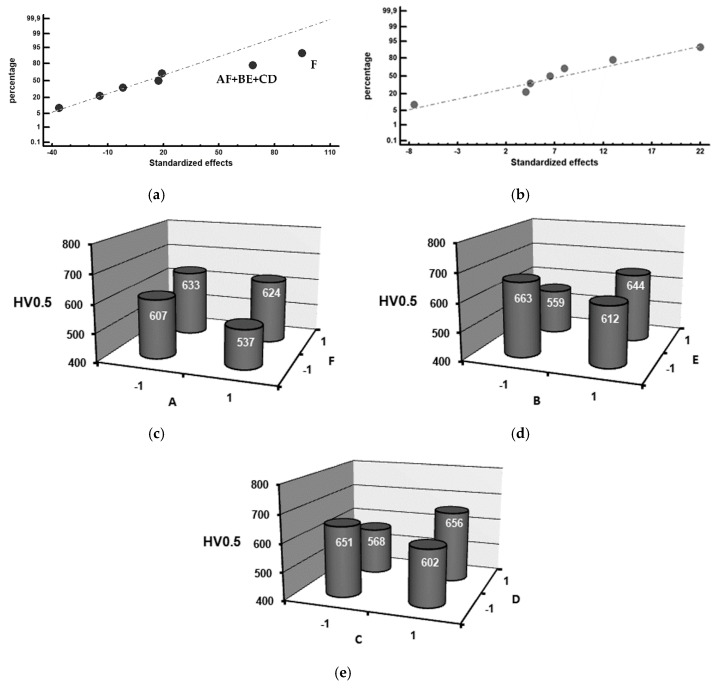
Factors that have a significant effect on hardness in the bond interface, (**a**) Standardized effects, on a normal probability plot, on the hardness in the shell region adjacent to the bond interface; (**b**) Standardized effects, on a normal probability plot, on the hardness in the core region adjacent to the bond interface; (**c**) Analysis of interaction AF; (**d**) Analysis of interaction BE; (**e**) Analysis of interaction CD.

Figure 2, Figure 3, Figure 4 and Figure 5 show the representation of these standardized effects on a normal probabilistic plot, highlighting those that have a significant effect on these responses.

Figure 2a shows that the factors with a significant effect on impact toughness are E (inoculation with SiCaMn) and A (inoculation with FeSi-La). Setting both factors at their respective +1 (0.6 kg/T SiCaMn) and −1 (without FeSi-La) levels leads to an increase in said toughness. In a previous study by the authors, it was found that inoculation with SiCaMn promoted discontinuity of the carbide network [32], which increased the toughness of the shell [29]. In this case, it is verified that this effect also reaches the region of the shell in contact with the core. In previous studies by the authors, it was similarly found that if the ladle is not inoculated with FeSi-La, then there is an improvement in the impact toughness inside the working layer [29,30,32]. In the present study, we have verified that this effect is maintained in the region of the shell-core bond. Previous studies [33,34] found that the aforementioned inoculant increases the number of graphite nodules and favors the precipitation of graphite with a lamellar morphology. If this inoculant should accumulate in the inner region of the working layer in contact with the core, it could damage the toughness of the shell-core bond. Given that FeSi-La has a density lower than the molten metal, it is suspected that FeSi-La tends to accumulate in the inner region of the shell during the centrifugation process. Figure 3a–c shows an example of this phenomenon. These micrographs show the region of the shell-core bond in a number of experiments in which FeSi-La was used as inoculant. The presence of flake graphite can be observed in these bonding regions.

It can also be seen that some of the interactions, namely AF, BE and CD, have a significant effect on toughness. Figure 2b through to Figure 2d shows the results of the analysis of these interactions. It appears that interaction BE, i.e., simultaneously setting both factors at their +1 level (inoculation with 6 kg/T FeB and 0.6 kg/T SiCaMn), produces an increase in toughness.

Figure 4 shows the hardness profiles obtained in the bonds in the eight experiments. It can be observed that there is a clear difference in hardness in the bond interface on the shell side between the different experiments. However, only minor differences are perceived on the side of the bond interface corresponding to the core. The statistically analyzed data correspond to a width of 2 mm in both regions.

Figure 5 shows the representation of standardized effects, on a normal probability plot, of the average hardness values in the bonding region. Figure 5a corresponds to the bonding region of the shell, while Figure 5b corresponds to the bonding region of the core. Mg is seen to have a significant influence on the hardness of the bonding region adjacent to the shell. Setting this factor at its +1 level (its addition up to 0.04 wt.%) produces an increase in hardness. This could be due to its lightness, its concentration increasing in the innermost region of the shell during its solidification by centrifugation. Mg produces high subcooling during eutectic solidification. Consequently, it shows a high tendency to form white eutectic versus its grey counterpart, especially if the Si content is below 2% [12]. None of the analyzed factors are found to have a significant effect on hardness in the core region adjacent to the bond interface. It can also be seen that some of the interactions, namely AF, BE and CD, have a significant effect on toughness. Figure 5c through to Figure 5e shows the results of the analysis of these interactions. In interaction AF, the significant effect of factor F can seemingly be seen. If this factor is set at its +1 level, increases in hardness in this interaction occur. For interaction BE, there appears to be an increase in hardness when these factors are simultaneously set at their −1 (recall the graphitizing effect of SiCaMn) and +1 (bear in mind the bleaching effect of FeB in this case) levels. Regarding interaction CD, this appears to produce an increase in hardness if the liquidus temperature (factor C) and %Si (factor D) are both at their −1 levels (this would be equivalent to a greater volume fraction of eutectic constituent and a lower fraction in graphite volume), or both at their +1 levels (a smaller volume fraction of the eutectic constituent and greater hardening due to the Si solid solution).

## 4. Conclusions

To enhance the quality of the shell-core interface in work rolls used in the finishing stands of hot strip mills, manufactured by indefinite chill double-poured centrifugal casting, it is advisable to:

1. Inoculate the shell (working layer) with 0.6 kg/T SiCaMn, as this promotes discontinuity in the carbide network and leads to an increase in the impact toughness of the bond interface.
2. Avoid inoculation with FeSi-La, as this inoculant promotes an increase in the number of graphite nodules per unit area and a lamellar morphology of this graphite, reducing the impact toughness in this bond interface.
3. Furthermore, the addition of Mg at values around 0.04 wt.% has been found to produce an increase in hardness in the shell region adjacent to the bond interface. This is due to two simultaneous phenomena:
  - The tendency of Mg to accumulate in the innermost region of the shell during centrifugal casting as a result of its low density.
  - Its tendency to favor the precipitation of white eutectic versus grey eutectic when the Si content is low.

## Figures and Tables

**Table 1:** Chemical composition range of the shell and the core (wt.%).

Part of the Roll	C	Si	Mn	Ni	Cr	Nb	Mo	Mg
Shell	3.2–3.4	0.9–1.0	0.8–1.0	4.4–4.6	1.7–1.8	0.65–0.75	0.25	-
Core	3–3.02	2.2–2.3	0.2–0.4	0.1–0.2	0–0.1	-	0–0.02	0.06–0.08

**Table 2:** Factors and levels analyzed in the design of experiments (DOE).

Factors			Levels	
Code	Description	Units	−1 Level	+1 Level
A	FeSi-La	wt.%	0	0.27
B	FeB	wt.%	0.3	0.6
C	Liquidus Temperature	K	1523–1528	1543–1548
D	Si	wt.%	0.8–0.85	1.1–1.15
E	SiCaMn	wt.%	0.03	0.06
F	Mg	wt.%	0	0.04

**Table 3:** Array of experiments.

No.	A	B	C	D	E	F	Confounding Pattern
1	−1	−1	−1	+1	+1	+1	Mean
2	+1	−1	−1	−1	−1	+1	A/BD/CE
3	−1	+1	−1	−1	+1	−1	B/AD/CF
4	+1	+1	−1	+1	−1	−1	C/AE/BF
5	−1	−1	+1	+1	−1	−1	D/AB/EF
6	+1	−1	+1	−1	+1	−1	E/AC/DF
7	−1	+1	+1	−1	−1	+1	F/BC/DE
8	+1	+1	+1	+1	+1	+1	AF/BE/CD

**Table 4:** Chemical composition of the inoculants used to manufacture the shell (wt.%). Remainder Fe.

Inoculants	Si	Ca	Al	Mn	Ti	Ba	C	Bi	S	P	B	La	Mg
FeSi-La	66.0	2.5	0.8	-	-	0.3	-	0.3	-	-	-	0.8	
FeSiMg	28.7	-	-	-	-	-	-	-	-	-	-	-	15.0
SiCaMn	58.3	16.4	1.1	14.8	0.03		0.6		0.03	0.03	-		
FeB	0.4	-	-	-	-		0.3		-	-	17.9		

**Table 5:** Casting parameters in each experiment. (wt.%)

Casting Parameters	Units	Experiment Number							
		1	2	3	4	5	6	7	8
**C**	%	3.35	3.46	3.4	3.28	2.94	3.04	3.02	3.04
Si	%	1.13	0.88	0.87	1.18	1.16	0.89	0.87	1.15
Mn	%	0.77	0.78	0.79	0.77	0.79	0.83	0.80	0.82
Ni	%	4.44	4.33	4.32	4.38	4.59	4.16	4.62	4.65
Cr	%	1.68	1.68	1.71	1.64	1.65	1.71	1.68	1.71
Mo	%	0.26	0.25	0.25	0.24	0.25	0.25	0.26	0.26
Mg	%	0.005	0.004	-	-	-	-	0.004	0.005
B	%	0.032	0.033	0.071	0.075	0.038	0.041	0.070	0.071
Nb	%	0.64	0.72	0.68	0.61	0.74	0.75	0.73	0.61
Liquidus Temperature	K	1525	1527	1526	1523	1546	1545	1545	1543

**Table 6:** Mean values and standardized effects. HV0.5 (Vickers hardness with a load of 0.5 kg).

Experiment	Charpy Test		Hardness of the Shell		Hardness of the Core		Confounding Pattern
	J/cm^2^	Effect	HV0.5	Effect	HV0.5	Values	
1	3.45	3.18	590	619.37	290	296.75	Mean
2	2.69	−0.36	707	−1.75	281	−7.50	A + BD + CE
3	3.66	0.13	595	17.25	313	22.00	B + AD + CF
4	2.57	0.18	547	19.25	294	4.50	C + AE + BF
5	3.26	0.11	619	−14.25	297	8.00	D + AB + EF
6	3.07	0.56	527	−36.25	275	6.50	E + AC + DF
7	3.09	0.09	677	94.75	302	4.00	F + BC + DE
8	3.69	0.28	693	68.25	322	13.00	AF + BE + CD
